# Effects of time-restricted eating on body composition and performance in athletes: a systematic review and meta-analysis

**DOI:** 10.3389/fnut.2026.1956159

**Published:** 2026-09-23

**Authors:** Ruting Lin, Yubo Wang, Zhenyu Shuai, Kaixiang Zhou, Wenqiang Wu

**Affiliations:** 1 College of Education, Beijing Sport University, Beijing, China; 2 Physical Education Department, College of Basic Education, Beijing College of Finance and Commerce, Beijing, China; 3 College of Physical Education and Health Science, Chongqing Normal University, Chongqing, China; 4 School of Strength Training and Conditioning, Beijing Sport University, Beijing, China

**Keywords:** athletes, body composition, Meta-analysis, performance, time-restricted eating

## Abstract

**Background:**

Time-restricted eating (TRE) has gained increasing attention as a dietary strategy for body mass management and metabolic health. However, its efficacy in athletic populations remains unclear. Therefore, this systematic review and meta-analysis aimed to examine the effects of TRE on energy intake, body composition, and performance in athletes.

**Methods:**

Following PRISMA guidelines, the review was registered in PROSPERO (CRD420261291351). Six databases were searched from inception to January 10, 2026. Randomized controlled trials comparing TRE with non-TRE dietary patterns in adult athletes were included if they reported body composition or performance outcomes. Pooled effects were estimated as standardized mean differences (SMDs; Hedges’ g) with 95% confidence intervals (CIs) using random-effects models. Risk of bias was assessed using RoB 2 and evidence certainty using GRADE.

**Results:**

Sixteen studies from 15 articles involving 245 athletes were included. TRE did not significantly alter energy intake (SMDpooled = −0.35, 95%CI: −0.77 to 0.07, *p* = 0.11). Compared with non-TRE dietary patterns, TRE resulted in small but significant reductions in body mass (SMDpooled = −0.27, 95%CI: −0.50 to −0.04; *p* = 0.02) and body fat% (SMDpooled = −0.39, 95%CI: −0.68 to −0.11, *p* = 0.01), while fat mass (SMDpooled = −0.23, 95%CI: −0.48 to 0.02, *p* = 0.07) and lean mass (SMDpooled = −0.16, 95%CI: −0.42 to 0.09, *p* = 0.21) were unchanged. TRE had no significant effects on aerobic capacity (SMDpooled = 0.04, 95%CI: −0.29 to 0.37, *p* = 0.82), muscular endurance (SMDpooled = −0.20, 95%CI: −0.52 to 0.13, *p* = 0.23), muscular strength (SMDpooled = 0.01, 95%CI: −0.25 to 0.27, *p* = 0.94), or power (SMDpooled = −0.12, 95%CI: −0.44 to 0.20, *p* = 0.47). Subgroup analyses suggested potentially greater reductions in body mass and body fat% among elite athletes, although between-subgroup differences were not statistically significant. Certainty of evidence ranged from very low to moderate.

**Conclusion:**

TRE may represent a feasible dietary strategy for athletes seeking modest reductions in body mass and body fat% without clear detrimental effects on performance. However, evidence remains limited by small samples, short interventions, and methodological concerns, warranting larger, well-controlled, and longer-term trials across diverse athletic populations.

**Systematic review registration:**

PROSPERO unique identifier CRD420261291351.

## 1 Introduction

In many sports, body composition management is an important component of performance preparation (1). This is particularly relevant in physique sensitive sports, weight category events, and endurance disciplines, as well as during preparatory phases when athletes seek to optimize competition readiness (1). However, achieving these goals through dietary restriction is inherently challenging, because reductions in energy intake may lower energy availability and thereby impair recovery and performance (2, 3). Accordingly, a key challenge in sports nutrition is to identify dietary strategies that support body composition management without compromising athletic performance.

Time-restricted eating (TRE) is now widely used as a practical dietary strategy for body weight management (4, 5). By restricting daily intake to a consistent eating window, typically 4–12 h, TRE has shown modest but consistent benefits for body mass and fat mass control in public health and clinical populations, together with improvements in selected metabolic outcomes (5–7). In athletes, adequate energy intake and nutrient timing support recovery and performance (8), whereas a fixed daily eating window may constrain nutrient intake around training and potentially compromise fueling (9). This potential trade-off between body-composition benefits and the maintenance of performance makes TRE particularly relevant in this population. Emerging evidence from exercise-based interventions suggests that TRE may support favorable changes in body composition without clear losses of lean mass or strength when combined with training (10–12). However, the available evidence in athletic populations remains inconsistent. For example, Correia et al. reported that a 4-week 16/8 TRE protocol did not significantly alter fat mass or body fat percentage (13), whereas Li et al. found that a 6-week 16/8 TRE intervention was associated with reductions in both fat mass and body fat% (14).

The purpose of this study was to evaluate whether TRE may represent a suitable dietary strategy for athletes by conducting a systematic review and meta-analysis of the available peer-reviewed literature on this topic. Specifically, we examined its effects on energy intake, body composition, and performance. Subgroup analyses were also conducted to explore whether these effects differed between recreational and elite athletes and to help inform future research. Our overarching hypothesis was that, relative to non-TRE dietary patterns, TRE would not substantially reduce total energy intake, but would improve body composition without compromising lean mass or performance.

## 2 Methods

### 2.1 Protocol and registration

This study was conducted following the Preferred Reporting Items for Systematic Reviews and Meta-Analyses (PRISMA) guidelines (15), and it has been registered in the PROSPERO database (CRD420261291351).

### 2.2 Data sources, searches, and study selection

Two authors (RL and YW) independently searched PubMed, Web of Science, Embase, Cochrane Library, Scopus, and EBSCO databases from inception to January 10, 2026, with no restriction on the earliest publication year. Search terms related to TRE and exercise were combined using the Boolean operators AND and OR; complete database-specific search strategies are provided in Supplementary Table S1. Reference lists of relevant publications were also manually searched.

Studies were eligible if they met the following PICOS criteria: (1) Population: athletes aged ≥18 years who regularly engaged in structured sport-specific training and/or competitive participation, with the aim of improving athletic performance (16, 17); (2) Intervention: time-restricted eating (TRE) was operationally defined as restricting daily caloric intake to an eating window of 8–12 h (4, 5). Related terminology, including time-restricted feeding, was considered TRE when the intervention met this definition; (3) Comparison: a non-TRE dietary pattern combined with the same or a comparable exercise program; (4) Outcomes: studies were required to report at least one outcome related to body composition or performance. Body composition outcomes included body mass, body fat, and lean mass. Performance outcomes included aerobic capacity (e.g., V̇O_2max_), muscular endurance (e.g., bench press muscular endurance), muscular strength (e.g., squat 1RM), and power (e.g., bench press rate of force development, squat jump height); (5) Study design: randomized controlled trials (RCTs) were included.

Studies were excluded if they met any of the following criteria: (1) Population: the participants were sedentary or insufficiently active individuals without regular exercise habits; (2) Intervention: the intervention was not TRE; (3) Comparison: the comparison was not a non-TRE dietary pattern combined with the same or a comparable exercise program; (4) Outcome: the study did not report at least one outcome related to body composition or performance; (5) Study design: review, conference articles, and animal trials; (6) Others: articles written in non-English languages and duplicate publications.

### 2.3 Data extraction, outcomes, and risk of bias assessment

The data extraction was carried out by two authors, RL and YW, while a third party, ZS, provided additional verification. The extracted data included details such as the authors, publication year, sample size, participant characteristics, dietary protocol, exercise protocol, intervention duration, and outcome measures. The mean and standard deviation of each outcome in the post-tests were extracted for every included study. If any relevant data were missing, we contacted the authors to request it (18). We obtained relevant data using WebPlotDigitizer (version 4.6) for studies when the authors could not be contacted for the data. Multiple reports were cross-checked for participant overlap. Exact duplicate outcomes from the same participant cohort were retained once, whereas non-identical effect estimates were retained.

Two investigators (RL and YW) independently assessed the risk of bias, resolving disagreements through discussion when possible or, if necessary, by arbitration from a third researcher, using the Cochrane Collaboration’s Risk of Bias Tool 2 (ROB2) (19). This tool evaluates five domains: randomization process, deviations from intended interventions, missing outcome data, measurement of the outcome, selection of the reported result.

### 2.4 Statistical analysis and grading the evidence

To assess the effect size (ES) of the intervention, we calculated the standardized mean difference (SMD; Hedges’g) of the outcomes (20), accompanied by a 95% confidence interval (CI). The effect size was categorized as trivial (<0.2), small (0.2 to 0.49), moderate (0.5 to 0.79), or large (>0.8) (21). A meta-analysis using the inverse variance method was conducted using Stata version 18.0 (STATA Corp., College Station, TX). The heterogeneity was evaluated by assessing the inconsistency (*I*^2^ statistic) of intervention effects across the trials. Heterogeneity quantification relied on *I*^2^ statistics, with values of 75, 50, and 25% indicating high, moderate, and low heterogeneity, respectively. A random-effects model was employed to estimate the pooled effect, considering the heterogeneity among studies due to participant variations and intervention characteristics. Publication bias was assessed using funnel plots and Egger’s test when at least 10 studies were available. If a significant asymmetry was detected, we used the Trim and Fill method to analyze the results. Meanwhile, we applied the Leave-One-Out method, which involved systematically removing individual studies to evaluate their impact on the overall results and to verify the robustness of the findings. Statistical significance was set at *p* < 0.05. Because some studies contributed multiple outcome indicators, two-level and three-level random-effects models were additionally compared in R (V.4.4.0; R Core Team, 2024) to assess potential dependency among effect sizes (22). To further assess robustness, outcome-specific robust variance estimation (RVE) was performed in Stata version 18.0 using robumeta when multiple correlated effect estimates arose from the same study or participant cohort. Additional sensitivity analyses excluded the long-term Moro assessment and, separately, Blake et al.

For subgroup analyses, participants were classified according to the Participant Classification Framework proposed by McKay et al. (16) into elite and recreational athletes. Elite athletes were operationally defined as Tier 3–5 participants, whereas recreational athletes were defined as Tier 1–2 participants.

Additionally, to evaluate the overall quality of evidence, we employed the GRADE (Grades of Recommendation, Assessment, Development, and Evaluation) approach. Eligible studies were assessed for risk of bias, imprecision, inconsistency, indirectness, publication bias, and other relevant factors. The overall quality of evidence was classified as high, moderate, low, or very low for each outcome (23, 24). Any disagreements between the two reviewers (RL and YW) were resolved through discussion, with input from a third reviewer (ZS) when necessary.

## 3 Results

This search identified 3,701 potentially relevant articles from PubMed (*n* = 1,006), Web of Science (*n* = 1864), Embase (*n* = 148), Cochrane Library (*n* = 486), Scopus (*n* = 83), and EBSCO (*n* = 114). After removing 895 duplicates, 2,617 records were excluded based on title and abstract. Consequently, 189 full-text articles were assessed for eligibility, of which 2 were unavailable. A further 173 studies were excluded for the following reasons: non-relevant outcome (*n* = 8), non-relevant interventions (*n* = 2), non-relevant participants (*n* = 126), and no full text (*n* = 37). In addition, we screened the reference lists of three published review articles and identified one additional study that met the inclusion criteria. A total of 16 studies (15 articles) were included in the final meta-analysis, and the PRISMA flow diagram is shown in Figure 1.

**Figure 1:**
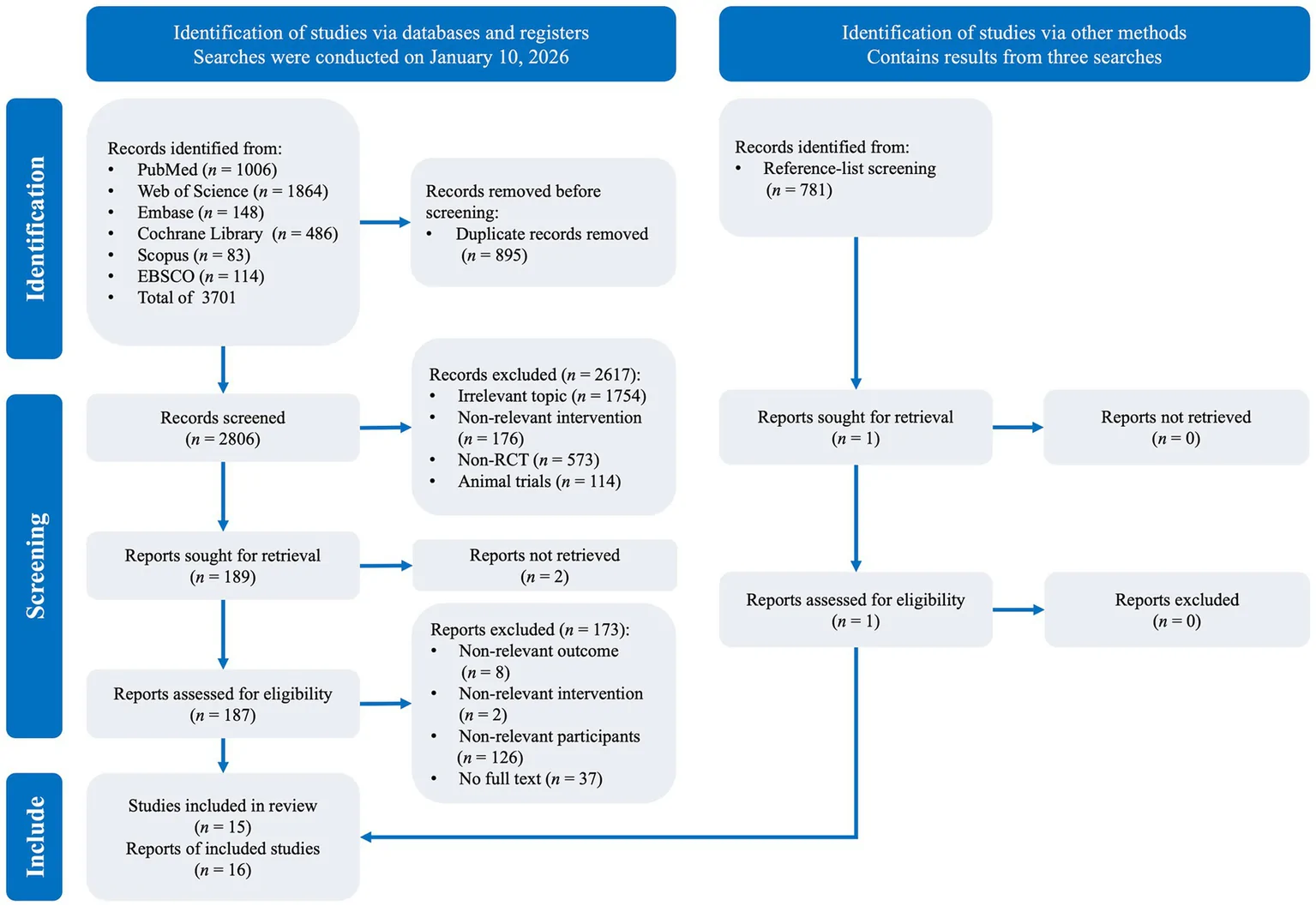
The PRISMA flow diagram.

### 3.1 Participant characteristics

A total of 245 participants were included in the analysis (Supplementary Table S2), including 202 men and 43 women, with mean ages ranging from 18.4 to 39.9 years. The study populations included resistance-trained athletes, runners, taekwondo athletes, and DanceSport athletes.

### 3.2 Dietary protocols

Most studies implemented a 16/8 TRE protocol, in which daily caloric intake was confined to an 8-h eating window followed by a 16-h fasting period. Only one study used a 12/12 protocol. When reported, eating windows generally began between 10:00 and 13:00 and ended between 18:00 and 21:00, although the exact timing varied across studies. In most studies, the control group followed a habitual, usual, or normal dietary pattern while undertaking a comparable exercise program. Dietary intake was managed differently across studies. Three studies imposed no additional caloric restriction or nutrition intervention (25–27). Two studies monitored food intake and compensated for identified energy deficits (14, 28). One study adjusted energy intake weekly to promote body mass gain during hypercaloric resistance training (29), whereas another prescribed a 25% caloric deficit in both groups (30). The remaining studies primarily manipulated meal timing while otherwise maintaining habitual or self-selected diets (13, 31–37).

### 3.3 Exercise protocols

Exercise protocols varied across studies and included resistance training, endurance running, and regular sport-specific training. Resistance training was the most common modality and was typically performed 3–4 times per week (29, 30, 36, 37). Endurance-based protocols mainly involved continuous or interval running (27, 28, 31, 33). Several studies included athletes who continued their habitual sport-specific training (14, 25). Exercise supervision also varied across studies, seven studies implemented supervised or structured training programs (13, 29, 30, 32, 34, 36, 37), whereas the remaining studies asked participants to maintain their habitual training routines or self-selected exercise programs. Intervention duration ranged from 4 to 52 weeks, although most studies lasted 4–8 weeks.

### 3.4 Outcome measurements

Fourteen studies evaluated body composition (13, 14, 26–37), most commonly using body mass, fat mass/body fat percentage, and lean mass/fat-free mass. Twelve studies assessed at least one performance outcome. Aerobic capacity was reported in five studies (13, 27, 28, 33, 34) and was primarily measured using VO_2max_ and VO_2peak_. Muscular endurance was evaluated in four studies (25, 29, 30, 36), mainly through repetitions to failure and taekwondo-specific kicking performance. Muscular strength was assessed in five studies (29, 30, 32, 36, 37) using 1RM tests and isometric force-related measures. Power was reported in six studies (25, 28, 30, 32, 35, 36), including countermovement jump height, squat jump height, and rate of force development.

### 3.5 Risk of bias

The overall risk of bias was assessed across five RoB2 domains (Figure 2). Eleven studies were judged as having some concerns (13, 14, 27, 29, 31–37), whereas four were rated as high risk of bias (25, 26, 28, 30). The randomization process showed the most favorable ratings, with 11 studies classified as low risk (13, 14, 27–30, 32, 34–37). By contrast, some concerns were frequently identified in the domains of deviations from intended interventions, missing outcome data, measurement of the outcome, and selection of the reported result. High-risk judgments were mainly driven by limitations in the randomization process, incomplete outcome data, and potential bias in outcome measurement.

**Figure 2:**
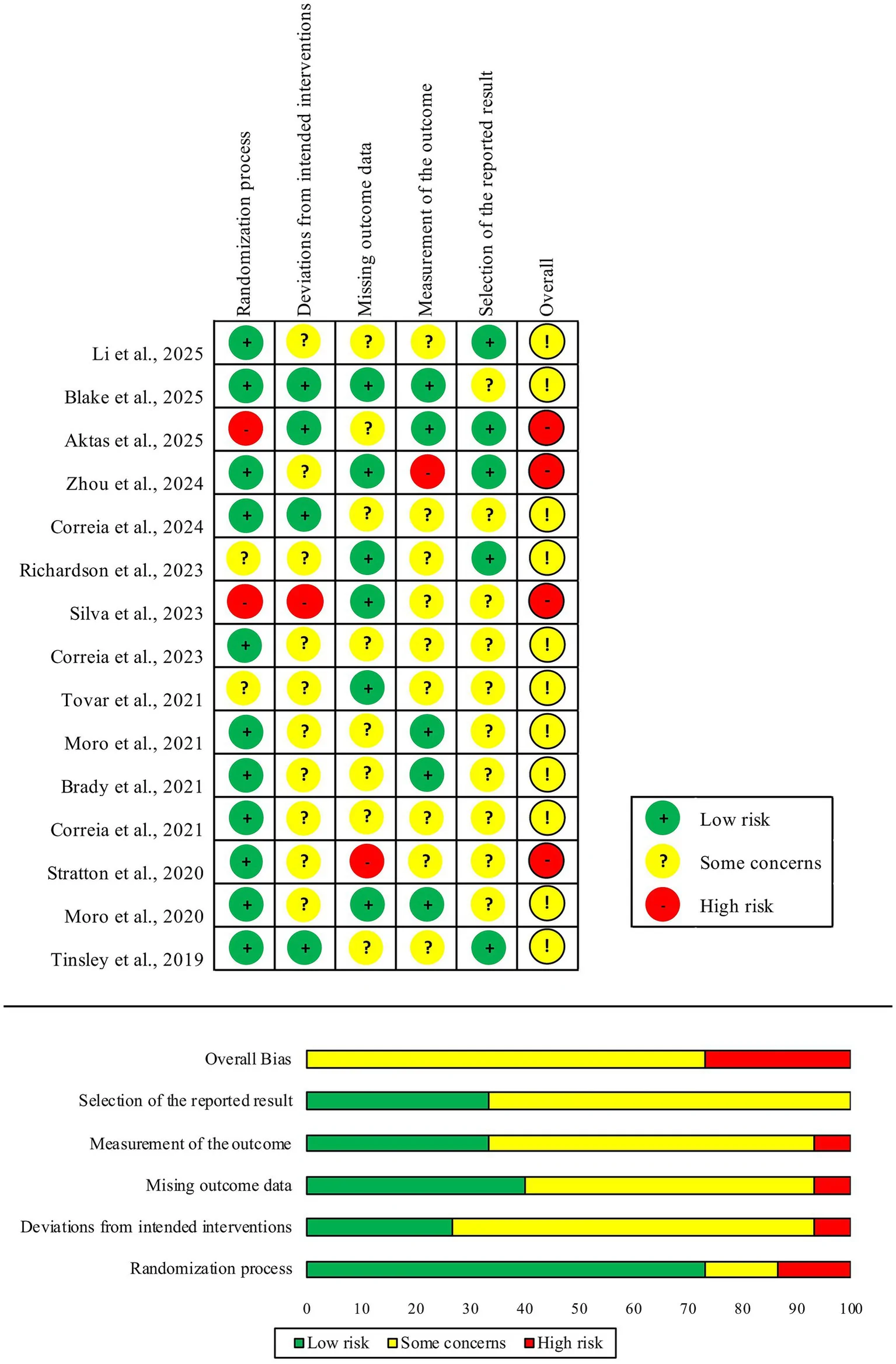
Risk of bias summary of the RoB2 assessments.

### 3.6 Meta-analysis

Comparison of the three-level model with reduced two-level models showed no deterioration in model fit (both LRT = 0.00, *p* = 1.00). Moreover, the reduced model yielded lower AIC and BIC values (AIC = 65.84, BIC = 70.72) than the three-level model (AIC = 67.84, BIC = 75.16), further supporting the simpler two-level specification. Accordingly, subsequent analyses were conducted using the two-level model.

#### 3.6.1 Energy intake

Six studies (14, 27, 28, 36, 37) reported changes in energy intake during the intervention, whereas the remaining studies either did not report these data or provided only overall mean values for the intervention as a whole.

##### 3.6.1.1 Energy intake

The pooled ES for energy intake was small and not significant (SMD_pooled_ = −0.35 [Hedges’g], 95%CI: −0.77 to 0.07, *p* = 0.11, low GRADE, Figure 3a), with no observed heterogeneity (*I^2^* = 0.00%, *p* = 0.58).

**Figure 3:**
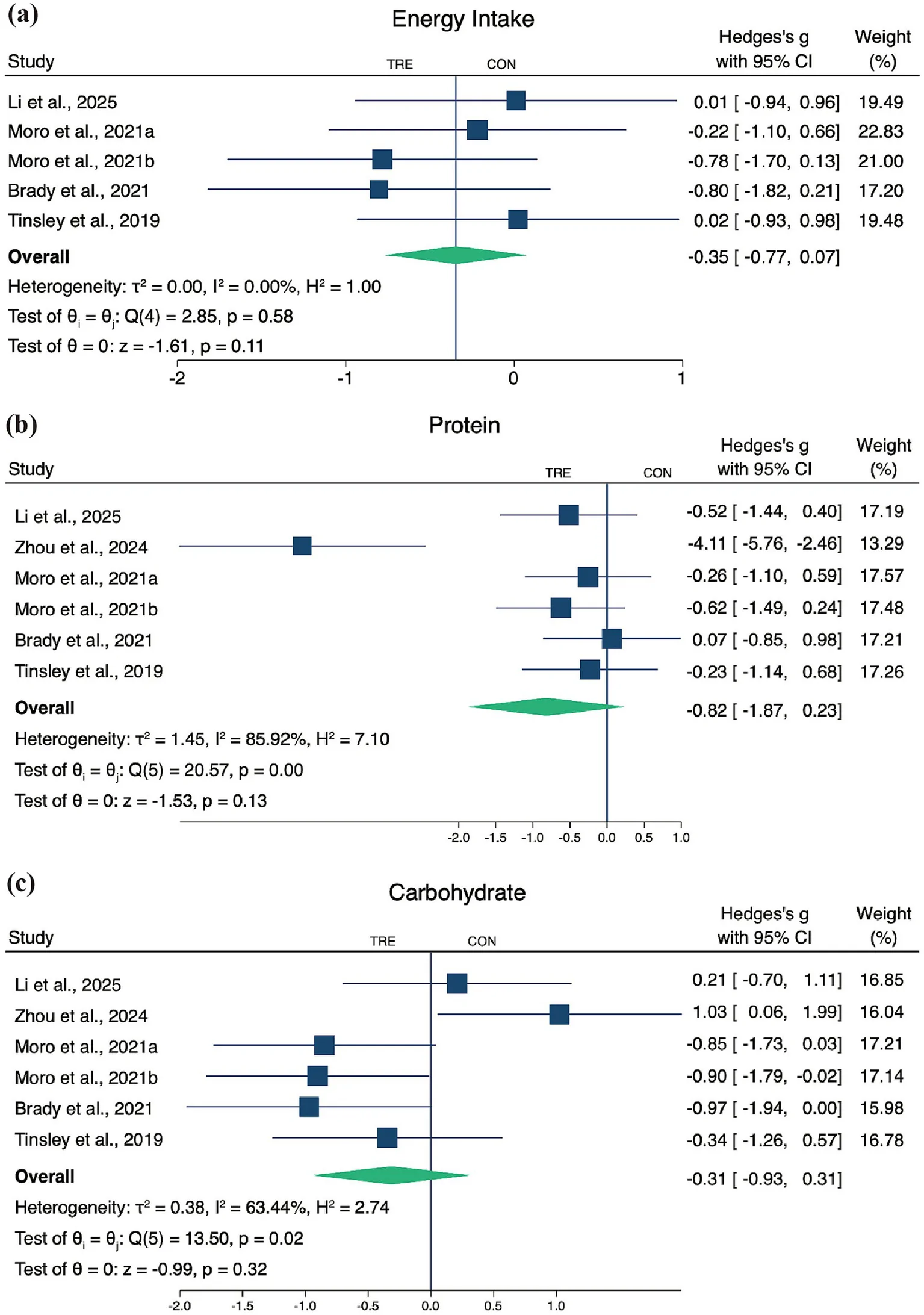
Forest plot of the effects of TRE on energy intake. **(a)** Energy intake, **(b)** Protein, **(c)** Carbohydrate.

##### 3.6.1.2 Protein

The pooled ES of protein was large and not significant (SMD_pooled_ = −0.82 [Hedges’g], 95%CI: −1.87 to 0.23, *p* = 0.13, very low GRADE, Figure 3b), with substantial heterogeneity (*I^2^* = 85.92%, *p* < 0.001).

##### 3.6.1.3 Carbohydrate

The pooled ES for carbohydrate was small and not significant (SMD_pooled_ = −0.31 [Hedges’g], 95%CI: −0.93 to 0.31, *p* = 0.32, very low GRADE, Figure 3c), with substantial heterogeneity (*I^2^* = 63.44%, *p* = 0.02).

#### 3.6.2 Effects of TRE on body composition

##### 3.6.2.1 Body mass

Four studies (26, 27, 34, 37) showed that TRE significantly reduced body mass compared with non-TRE dietary patterns, whereas the remaining studies reported no significant between-group differences.

The pooled ES of body mass was small and significant (SMD_pooled_ = −0.27 [Hedges’g], 95%CI: −0.50 to −0.04, *p* = 0.02, low GRADE, Figure 4), without heterogeneity (*I^2^* = 0.00%, *p* = 0.91). The funnel plot (Supplementary Figure S1) and Egger’s test (*t* = 0.46, *p* = 0.65) indicated no publication bias.

**Figure 4:**
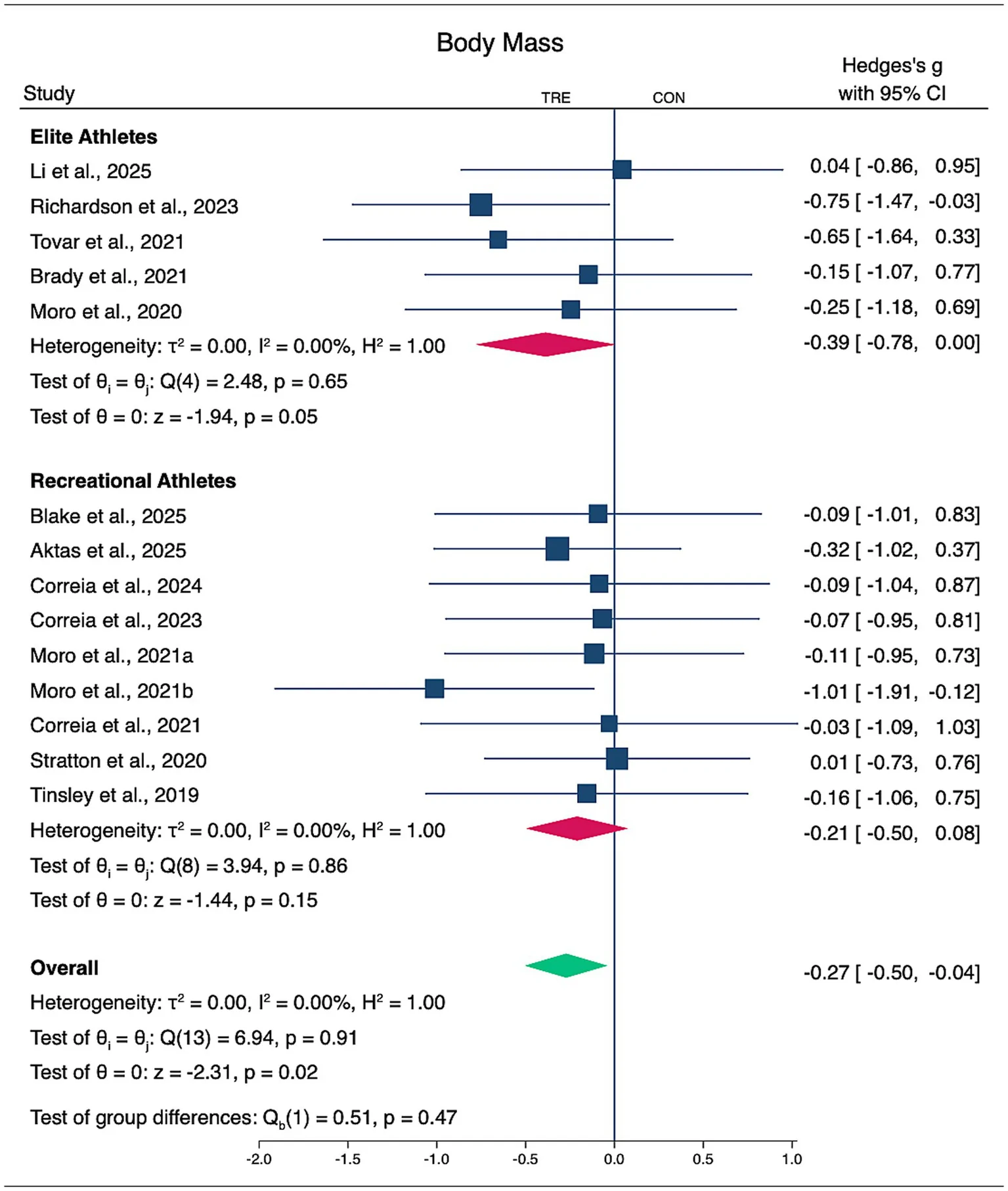
Forest plot of the effects of TRE on body mass.

Subgroup analysis suggested a larger reduction in body mass among elite athletes than among recreational athletes, although the between-subgroup difference was not statistically significant. In elite athletes, the pooled ES was small and borderline significant (SMD_pooled_ = −0.39 [Hedges’g], 95%CI: −0.78 to 0.00, *p* = 0.05, Figure 4), whereas no significant effect was observed in recreational athletes (SMD_pooled_ = −0.21 [Hedges’g], 95%CI: −0.50 to 0.08, *p* = 0.15, Figure 4).

##### 3.6.2.2 Fat mass

Seven studies (14, 26, 28, 29, 31, 33, 37) reported a significant reduction in fat mass compared with non-TRE dietary patterns, whereas the remaining studies reported no significant between-group differences.

The pooled ES for fat mass was small and not significant (SMD_pooled_ = −0.23 [Hedges’g], 95%CI: −0.48 to 0.02, *p* = 0.07, low GRADE, Figure 5), with no observed heterogeneity (*I^2^* = 0.00%, *p* = 1.00). The funnel plot (Supplementary Figure S1) and Egger’s test (*t* = 0.34, *p* = 0.74) indicated no publication bias. The RVE sensitivity analysis yielded a similar point estimate, although the 95% CI excluded the null (SMD_pooled_ = −0.22 [Hedges’g], 95%CI: −0.32 to −0.12, *p* < 0.001, Supplementary Table S6).

**Figure 5:**
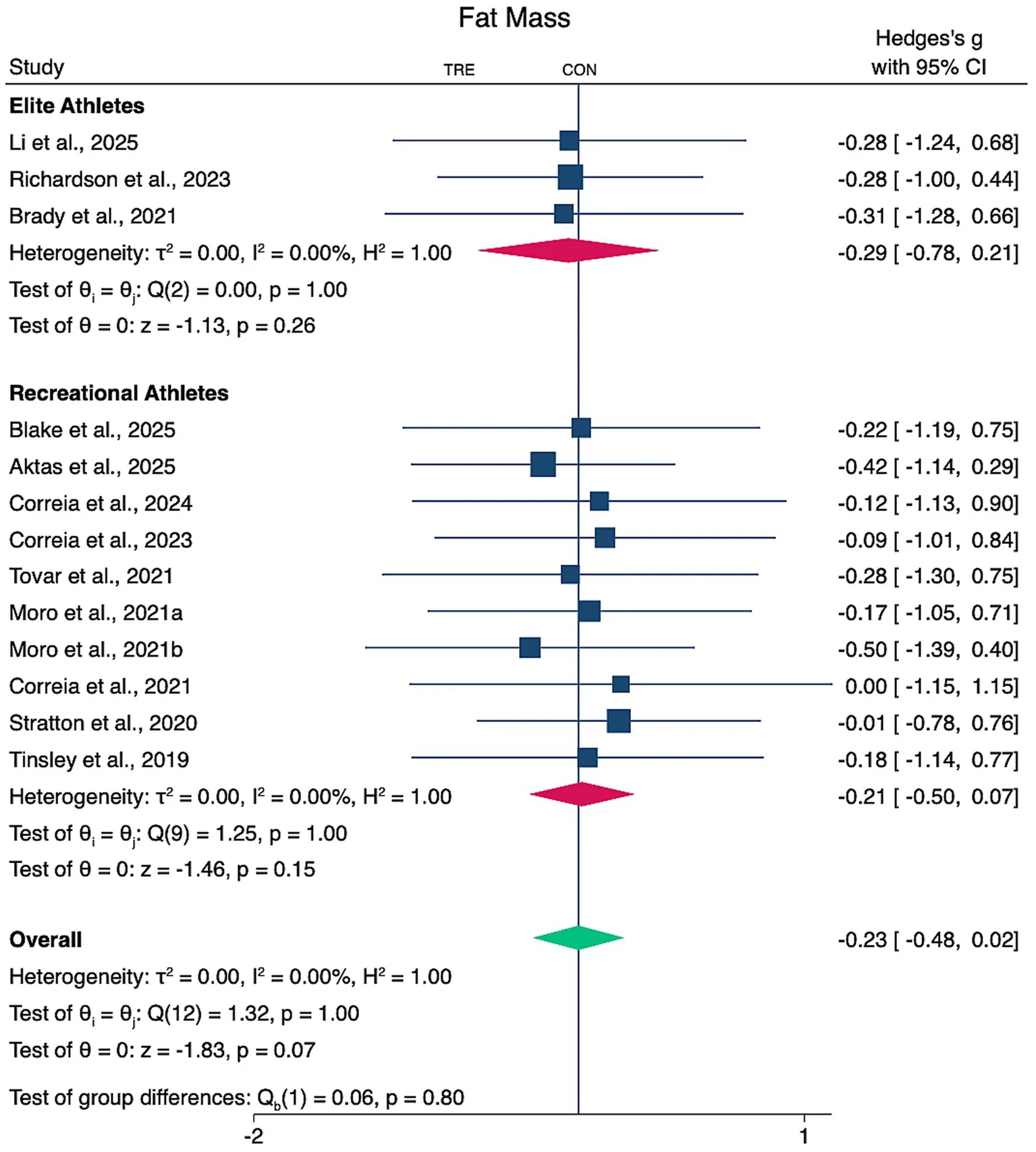
Forest plot of the effects of TRE on fat mass.

Subgroup analysis revealed no statistically significant difference in the effect of TRE on fat mass between elite athletes and recreational athletes. Specifically, the pooled effect sizes were small and non-significant in both elite athletes (SMD_pooled_ = −0.29[Hedges’g], 95%CI: −0.78 to 0.21, *p* = 0.26) and recreational athletes (SMD_pooled_ = −0.21[Hedges’g], 95% CI −0.50 to 0.07, *p* = 0.15).

##### 3.6.2.3 Body fat%

Six studies (14, 26, 28, 29, 31, 33) reported a significant reduction in body fat% compared with non-TRE dietary patterns, whereas the remaining studies reported no significant between-group differences.

The pooled ES of body fat% was small and significant (SMD_pooled_ = −0.39 [Hedges’g], 95%CI: −0.68 to −0.11, *p* = 0.01, moderate GRADE, Figure 6), without heterogeneity (*I^2^* = 0.00%, *p* = 0.92). The funnel plot (Supplementary Figure S1) and Egger’s test (*t* = 0.40, *p* = 0.70) indicated no publication bias.

**Figure 6:**
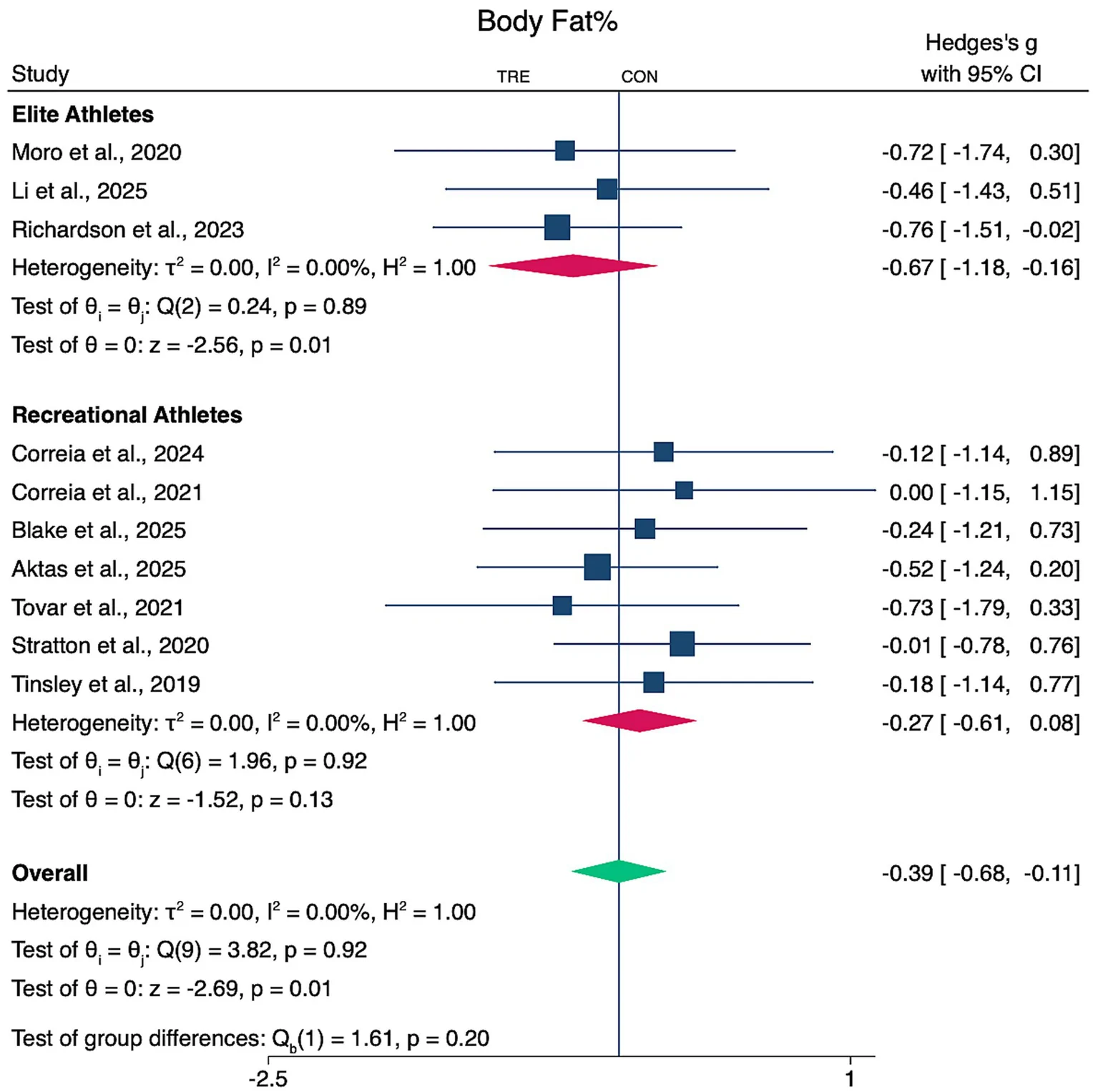
Forest plot of the effects of TRE on body fat%.

Subgroup analysis indicated a larger reduction in body fat percentage among elite athletes than among recreational athletes, although the between-subgroup difference was not statistically significant. In elite athletes, the ES was moderate and significant (SMD = −0.67 [Hedges’g], 95%CI: −1.18 to −0.16, *p* = 0.01), whereas in recreational athletes it was small and non-significant (SMD = −0.27 [Hedges’g], 95%CI: −0.61 to 0.08, *p* = 0.13).

##### 3.6.2.4 Lean mass

Two studies (26, 37) reported a significant reduction in lean mass compared with non-TRE dietary patterns, whereas the remaining studies reported no significant between-group differences.

The pooled ES of lean mass was trivial and not significant (SMD_pooled_ = −0.16 [Hedges’g], 95%CI: −0.42 to 0.09, *p* = 0.21, low GRADE, Figure 7) with no observed heterogeneity (*I^2^* = 0.00%, *p* = 0.99). The funnel plot (Supplementary Figure S1) and Egger’s test (*t* = 0.83, *p* = 0.42) indicated no publication bias. The RVE sensitivity analysis yielded a similar point estimate, although the 95% CI excluded the null (SMD_pooled_ = −0.14 [Hedges’g], 95%CI: −0.27 to −0.01, *p* = 0.03, Supplementary Table S6).

**Figure 7:**
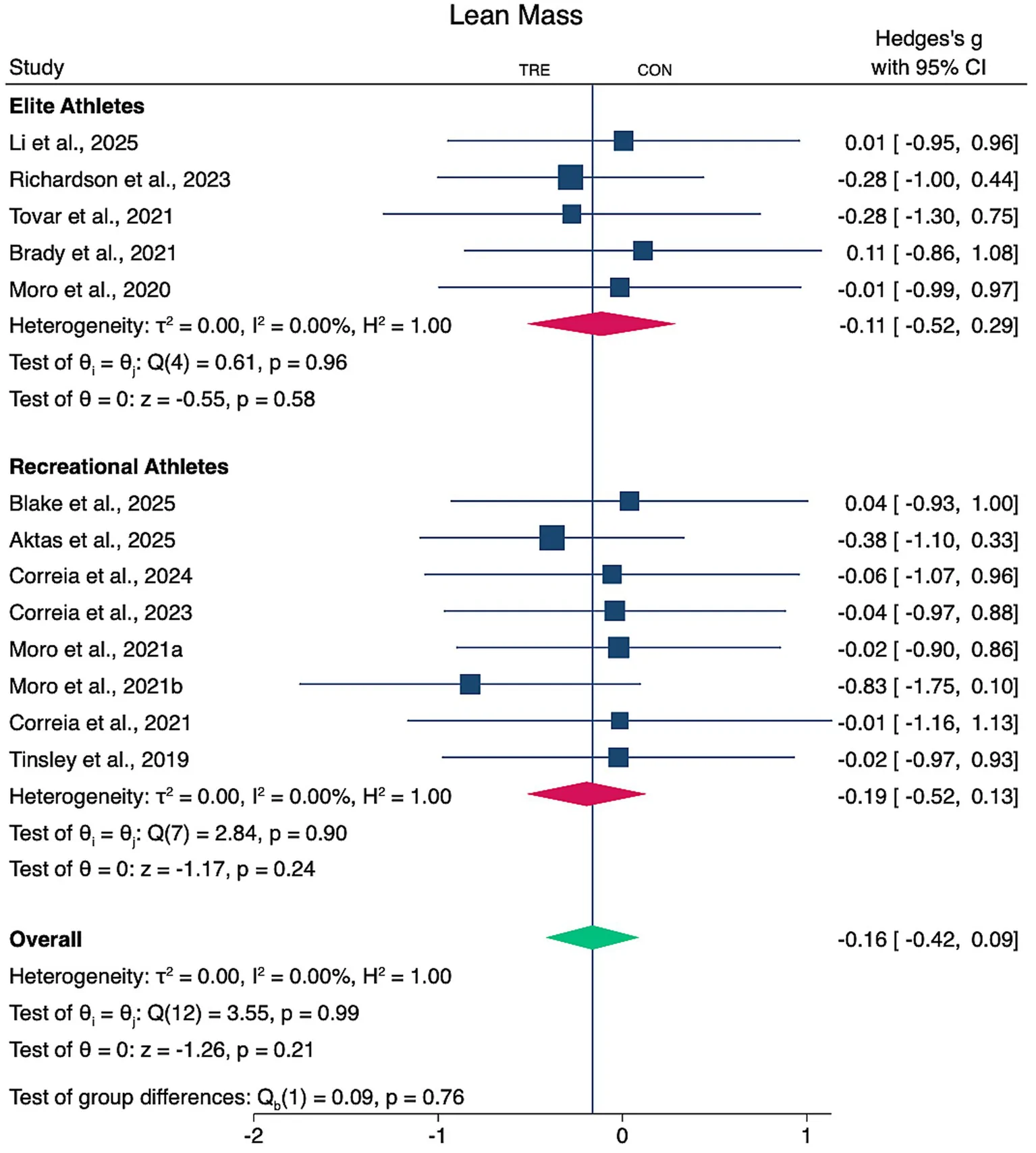
Forest plot of the effects of TRE on lean mass.

Subgroup analysis revealed no statistically significant difference in the effect of TRE on lean mass between elite athletes and recreational athletes. Small and non-significant effects were observed in both elite athletes (SMD_pooled_ = −0.11 [Hedges’g], 95%CI: −0.52 to 0.29, *p* = 0.58) and recreational athletes (SMD_pooled_ = −0.19 [Hedges’g], 95%CI: −0.52 to 0.13, *p* = 0.24).

#### 3.6.3 Effects of TRE on performance

##### 3.6.3.1 Aerobic capacity

None of the included studies reported a significant between-group difference in aerobic capacity between the TRE and non-TRE groups.

The pooled ES of aerobic capacity was trivial and not significant (SMD_pooled_ = 0.04 [Hedges’g], 95%CI: −0.29 to 0.37, *p* = 0.82, low GRADE, Figure 8a) without heterogeneity (*I^2^* = 0.00%, *p* = 0.78).

**Figure 8:**
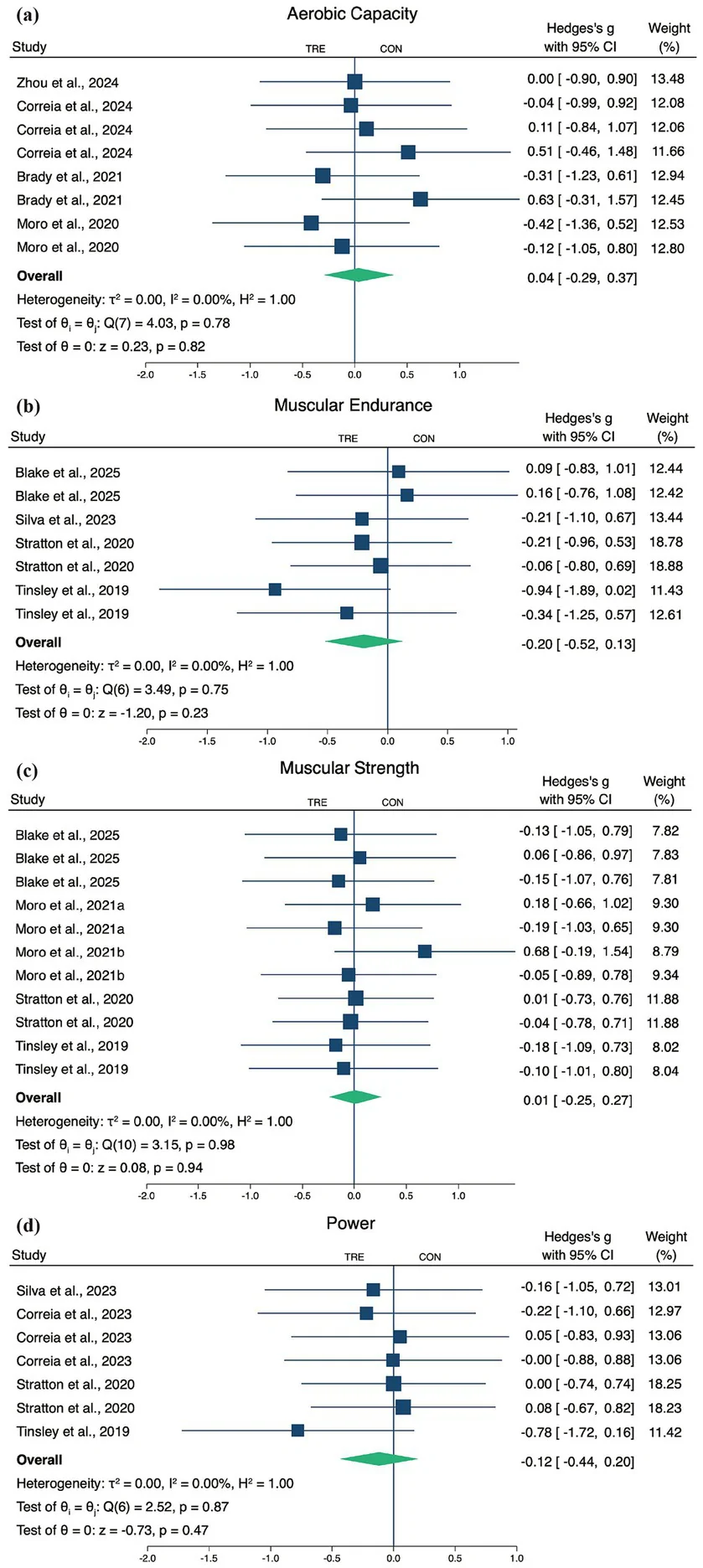
Forest plot of the effects of TRE on performance. **(a)** Aerobic capacity, **(b)** Muscular endurance, **(c)** Muscular strength, **(d)** Power.

##### 3.6.3.2 Muscular endurance

None of the included studies showed a significant improvement in muscular endurance with TRE compared with non-TRE dietary patterns.

The pooled ES of muscular endurance was small and not significant (SMD_pooled_ = −0.20 [Hedges’g], 95%CI: −0.52 to 0.13, *p* = 0.23, low GRADE, Figure 8b) without heterogeneity (*I^2^* = 0.00%, *p* = 0.75).

##### 3.6.3.3 Muscular strength

None of the included studies showed a significant improvement in muscular strength with TRE compared with non-TRE dietary patterns.

The pooled ES of muscular strength was trivial and not significant (SMD_pooled_ = 0.01 [Hedges’g], 95%CI: −0.25 to 0.27, *p* = 0.94, low GRADE, Figure 8c) without heterogeneity (*I^2^* = 0.00%, *p* = 0.98). The funnel plot (Supplementary Figure S1) and Egger’s test (*t* = −0.13, *p* = 0.90) indicated no publication bias.

##### 3.6.3.4 Power

One study (32) reported a significant improvement in power compared with non-TRE dietary patterns, whereas findings from the remaining studies did not consistently favor TRE.

The pooled ES of power was trivial and not significant (SMD_pooled_ = −0.12 [Hedges’g], 95%CI: −0.44 to 0.20, *p* = 0.47, low GRADE, Figure 8d) with no heterogeneity (*I^2^* = 0.00%, *p* = 0.87).

### 3.7 GRADE assessment and sensitivity analyses

The GRADE assessment showed very low to moderate certainty evidence across all outcomes, with downgrading primarily due to risk of bias and, for several outcomes, further reductions due to imprecision (Supplementary Table S7). Sensitivity analyses examining the exclusion of the long-term Moro assessment and Blake et al., as well as outcome-specific RVE, are presented in Supplementary Tables S5, S6.

## 4 Discussion

This systematic review and its meta-analysis provide very low- to moderate-certainty evidence that TRE may improve body composition in athletes without clear impairment of performance. Specifically, TRE was associated with reductions in body mass and body fat%, whereas fat mass, lean mass and total energy intake did not differ significantly from non-TRE dietary patterns. Subgroup analyses showed numerically larger estimates for body mass and body fat% among elite athletes, although between-subgroup differences were not statistically significant. These findings may inform practical dietary strategies for athletes seeking to optimize body composition while preserving performance.

### 4.1 Effects of TRE on body composition

The present findings indicate that TRE reduces body mass and body fat% in athletes without significantly affecting lean mass. These results extend previous evidence from overweight and obese populations to athletes, supporting the potential applicability of TRE beyond sedentary groups (7, 10). One plausible explanation is that concentrating intake within a narrower daily window may alter nutrient timing, prolong the post absorptive period, and improve alignment between feeding behavior and circadian biology, thereby favoring fat oxidation and metabolic efficiency (38, 39). Experimental work by Hatori et al. further suggests that time restricted feeding may influence CREB, mTOR, and AMPK signaling, restore circadian oscillations, and enhance nutrient utilization and energy expenditure, providing a plausible mechanistic basis for the reductions in body mass and adiposity observed in the present analysis (40).

Importantly, no significant difference in lean mass was observed despite concurrent reductions in body mass and body fat%. This may be partly explained by the anabolic support provided by ongoing resistance training together with adequate protein intake (41, 42). Consistent with this interpretation, Tinsley et al. reported that an 8 week 16/8 time restricted feeding intervention combined with supervised resistance training and whey protein supplementation did not reduce fat free mass in females with resistance training experience relative to a control diet (36). By contrast, Cui et al. found that, in physically active young adults with overweight or obesity, a 10 week 10 h TRE intervention reduced fat free mass when implemented without resistance training, whereas the addition of resistance training attenuated this response and was more favorable for maintaining muscle mass (43). However, several included studies did not adequately account for total energy intake, which may partly explain the variability in lean mass responses. The RVE sensitivity analysis also yielded a similar negative point estimate with a 95% CI excluding the null, indicating some uncertainty regarding this outcome. Future studies in athletes should therefore adopt more clearly standardized dietary and exercise protocols, particularly with respect to protein intake, to better define the conditions under which TRE can facilitate body mass control while preserving lean mass.

### 4.2 Effects of TRE on performance

We also found that TRE did not significantly affect aerobic capacity, muscular endurance, muscular strength, or power, suggesting that, under the conditions examined, TRE does not appear to meaningfully impair performance in athletes. One possible explanation is that the body-composition changes induced by TRE were modest; although body mass and body fat% were significantly reduced, the magnitude of these effects was small and may have been insufficient to alter the key physiological determinants of performance (1). The absence of a decline in lean mass may also have contributed to the maintenance of performance, particularly for outcomes such as muscular strength, endurance, and power that depend heavily on lean tissue and neuromuscular function (44). In addition, the continued training stimulus across the included studies may have offset any potential negative effects of a compressed eating window, as regular training helps preserve metabolic adaptation, neuromuscular function, and sport-specific capacity (36, 42). From the perspective of energy availability, these findings further suggest that, although TRE may theoretically increase the risk of low energy availability, it does not necessarily translate into measurable performance impairment, at least in the short term and under the dietary and training conditions examined here.

This interpretation is supported by several individual trials. Tovar et al. reported that a 4-week 16/8 TRE intervention in endurance-trained male runners reduced fat mass while maintaining fat-free mass and running performance (33). Similarly, Zhou et al. found that a 6-week TRE intervention in female DanceSport dancers improved body composition without compromising performance (28). Brady et al. likewise observed that an 8-week 16:8 TRE intervention in trained male middle- and long-distance runners did not alter indices of endurance running performance (27). Collectively, these findings suggest that TRE may be a feasible strategy for body-composition management without obvious short-term performance trade-offs, which may be particularly relevant for female DanceSport dancers and athletes in weight-category sports who require body mass control (14, 25, 28). However, because most included interventions were short in duration and the certainty of evidence ranged from very low to moderate, longer term and better controlled trials are needed to determine whether these findings remain stable over time and across different training contexts.

## 5 Limitations

Several limitations in the current analysis should be noted. First, the available evidence base was small, with a total sample of 245 participants, which limits the precision and generalizability of the pooled estimates. Second, most interventions were short in duration, typically lasting 4 to 8 weeks, so the present findings mainly reflect the short-term effects of TRE rather than its longer-term applicability in athletes. Finally, several studies did not adequately control or report total energy and macronutrient intake, making it difficult to distinguish the effects of meal timing from those of concurrent changes in energy balance and dietary composition. In addition, variation in eating-window duration and timing may have affected between-study comparability, and some findings were sensitive to the treatment of correlated effect estimates.

## 6 Conclusion

The findings of this review indicate that TRE may have potential as a practical dietary strategy for achieving modest reductions in body mass and body fat% without clear detrimental effects on performance in athletes. Subgroup analyses suggested potentially greater reductions in body mass and body fat% among elite athletes, although between-subgroup differences were not statistically significant. Given the small sample sizes and short intervention durations, further well-controlled and longer-term trials are needed to clarify the effects of TRE on body composition and performance in athletes.

## Data Availability

The original contributions presented in the study are included in the article/Supplementary material, further inquiries can be directed to the corresponding author.
