# Correction: Effects of time-restricted eating on body composition and performance in athletes: a systematic review and meta-analysis

**DOI:** 10.3389/fnut.2026.2010386

**Published:** 2026-10-26

**Authors:** Ruting Lin, Yubo Wang, Zhenyu Shuai, Kaixiang Zhou, Wenqiang Wu

**Affiliations:** 1 College of Education, Beijing Sport University, Beijing, China; 2 Physical Education Department, College of Basic Education, Beijing College of Finance and Commerce, Beijing, China; 3 College of Physical Education and Health Science, Chongqing Normal University, Chongqing, China; 4 School of Strength Training and Conditioning, Beijing Sport University, Beijing, China

**Keywords:** athletes, body composition, Meta-analysis, performance, time-restricted eating

The equal-contribution symbol (†) for authors Ruting Lin and Yubo Wang was inadvertently omitted from the published article. Ruting Lin and Yubo Wang contributed equally to this work and share first authorship.

The original version of this article has been updated.

