# Supplementary material for: Effects of time-restricted eating on body composition and performance in athletes: a systematic review and meta-analysis
Source: Front Nutr. 2026 Sep 23;13:1956159. doi: 10.3389/fnut.2026.1956159 (PMC13644690; doi:10.3389/fnut.2026.1956159)
Supplement: Supplementary file 1 [file Data_Sheet_1.docx]

Supplementary Material

# 1 Supplementary Method

## 1.1 Search Strategy

Table S1 Complete search strategy for each database

| Database | Query | Results |
| --- | --- | --- |
| PubMed | (time-restricted feeding OR time-restricted eating OR intermittent fasting OR time-restricted eating OR time-restricted diet OR time-restricted meal) AND (exercise OR "physical activity" OR training OR fitness OR running OR cycling)  Filters: Full text, Humans | 1006 |
| Web of  Science | (ALL=(time-restricted feeding OR time-restricted eating OR intermittent fasting OR time-restricted eating OR time-restricted diet OR time-restricted meal)) AND (ALL=(exercise OR "physical activity" OR training OR fitness OR running OR cycling)) | 1864 |
| Embase | (time-restricted feeding OR time-restricted eating OR intermittent fasting OR time-restricted eating OR time-restricted diet OR time-restricted meal) AND (exercise OR "physical activity" OR training OR fitness OR running OR cycling)  Filters: Humans | 148 |
| Cochrane  Library | (time-restricted feeding OR time-restricted eating OR intermittent fasting OR time-restricted eating OR time-restricted diet OR time-restricted meal) in Title Abstract Keyword AND (exercise OR "physical activity" OR training OR fitness OR running OR cycling) in Title Abstract Keyword  In Trials (Word variations have been searched) | 486 |
| Scopus | (time-restricted feeding OR time-restricted eating OR intermittent fasting OR time-restricted eating OR time-restricted diet OR time-restricted meal) in Title Abstract Keyword AND (exercise OR "physical activity" OR training OR fitness OR running OR cycling) in Title Abstract Keyword | 83 |
| EBSCO | (time-restricted feeding OR time-restricted eating OR intermittent fasting OR time-restricted eating OR time-restricted diet OR time-restricted meal) AND (exercise OR "physical activity" OR training OR fitness OR running OR cycling)  Filters: Full text | 114 |

# 2 Supplementary Result

## 2.1 Characteristics of the studies

Table S2 Characteristics of studies and effect sizes included in the meta-analysis

| **N.** | **Study** | **Study type; setting** | **Participants N, M/F** | **Age; BMI** | **Eligibility criteria** | **Exercise (frequency, intensity, time, type)** | **Dietary strategy** | **Adherence** | **Control group** | **Outcome** |
| --- | --- | --- | --- | --- | --- | --- | --- | --- | --- | --- |
|  |  |  |  |  |  |  |  |  |  |  |
| 1 | Li et al., 2025 | RCT | N=17, 0/17 | EXP: 19±0.94years; 19.53±1.95kg/m² CON: 19.5±1.43years; 18.89±1.56kg/m² | elite DanceSport athletes | Duration: 6 weeks | Protocol: 16 (fasting) / 8 (eating) | Three participants (one from the TRE group and two from the Control group) were unable to complete the posttest measurements for blood indices due to uncontrollable. circumstances. | Habitual dietary patterns | BM→  FFM →  FM ↓  BF% ↓ |
|  |  |  |  |  |  |  | Eating window: 11:00 AM to 7:00 PM |  |  |  |
|  |  |  |  |  |  |  | Other: food intake was recorded and converted into caloric values, participants with an identified energy deficit were instructed to compensate on Sundays by choosing foods that provided the necessary caloric intake. |  |  |  |
| 2 | Blake et al., 2025 | RCT | N=17, 10/7 | EXP(M): 25.8±3.9years; 25.9 ± 2.0kg/m² CON(F): 22.8±2.2years; 24.4 ± 0.3kg/m² CON(M): 25.1±3.0years; 26.6 ± 2.0kg/m² EXP(F): 24.2±1.4years; 22.6 ± 1.7kg/m² | well-trained recreational athletes | F: 4 times/week (alternating between two workouts (A-B-A-B)) | Protocol: 16 (fasting) / 8 (eating) | Adherence to the nutrition (eating windows, calories, and protein) and exercise protocols were >94% in both groups. | Performed RE before noon but consumed all daily macronutrients throughout the day at their discretion | Squat 1RM ↓  Bench Press 1RM →  Bench Press ME →  Leg Extension 1RM →  Leg Press ME →  BM →  FFM →  FM ↓  BF% ↓ |
|  |  |  |  |  |  | I: as many repetitions as possible without failing any repetitions | Other: If a participant’s average weekly body weight did not increase by >1%, energy intake was increased by 10% before beginning the next week of training. |  |  |  |
|  |  |  |  |  |  | T: / |  |  |  |  |
|  |  |  |  |  |  | T: autoregulatory progressive resistance exercise (APRE) |  |  |  |  |
|  |  |  |  |  |  | Duration: 8 weeks |  |  |  |  |
| 3 | Aktas et al., 2025 | RCT | N=31, 31/0 | EXP: 26.7±7.2years; 78.1±5.6kg/m² CON: 28.3±4.8years; 74.8±11.1kg/m² | Recreational-level athletes in cycling, running, and/or soccer | Duration: 6 weeks | Protocol: 16 (fasting) / 8 (eating) | Two participants from the TRE group withdrew from the study due to personal reasons, including relocating to another city and changes in physical activity habits. | Maintain eating habits | BM ↓  FM ↓  LM ↓  BF% ↓ |
|  |  |  |  |  |  |  | Eating window: 10:00 AM to 6:00 PM or 11:00 AM to 7:00 PM |  |  |  |
| 4 | Zhou et al., 2024 | RCT | N=17, 0/17 | EXP: 19±0.94years; 19.53±1.95kg/m² CON: 19.5±1.43years; 18.89±1.56kg/m² | elite DanceSport athletes | Duration: 6 weeks | Protocol: 16 (fasting) / 8 (eating) | Three participants (one from the TRE group and two from the ND) were unable to complete the posttest measurements for blood indices due to uncontrollable. circumstances. | Habitual dietary patterns | FM ↓  FFM →  FM% ↓  VO2max → |
|  |  |  |  |  |  |  | Eating window: 11:00 AM to 7:00 PM |  |  |  |
|  |  |  |  |  |  |  | Other: Participants with an energy deficit were instructed to supplement their lack of energy every Sunday by choosing foods containing suitable calories. |  |  |  |
| 5 | Correia et al., 2024 | RCT | N=15, 15/0 | ALL: 23.7±2.6years; / | Recreational runners | F: 3 10km runs/week(week1-2); 3 running bouts of 1 km (following each continuous run, week3-4) | Protocol: 16 (fasting) / 8 (eating) | One participant was excluded 1 week after entering the study because of a lack of adherence to the study protocol. | Habitual dietary patterns | BM →  FMs →  FM% →  FFM →  VO2max → |
|  |  |  |  |  |  | I: 10km (VT1-VT2); 1km (VT2-VO2max) | Eating window: 1:00 PM to 9:00 PM |  |  |  |
|  |  |  |  |  |  | T: / |  |  |  |  |
|  |  |  |  |  |  | T: continuous runs and interval running |  |  |  |  |
|  |  |  |  |  |  | Duration: 4 weeks |  |  |  |  |
| 6 | Richardson et al., 2023 | cross-over study | N=15, 15/0 | ALL: 28.7±5.2years; / | Highly trained male runners | Duration: 4 weeks | Protocol: 16 (fasting) / 8 (eating) | Four subjects dropped out after the familiarization visit due personal commitments. Five subjects did not complete both arms of the study due to personal commitments (n = 4) or early cessation of data collection related to COVID-19 restrictions (n =1). | Normal dietary pattern(12/12; 12h eating/fasting times) | BM→  FM ↓  FFM →  BF% ↓ |
| 7 | Silva et al., 2023 | experimental paired trial | N=9, 7/2 | ALL: 18.4±3.3years; / | Elite athletes | F: 6 sessions/week | Protocol: 12 (fasting) / 12 (eating) | / | Maintain their eating habits | CMJ ↓ |
|  |  |  |  |  |  | I: normal training | Other: no nutrition intervention was performed |  |  |  |
|  |  |  |  |  |  | T: / |  |  |  |  |
|  |  |  |  |  |  | T: Taekwondo |  |  |  |  |
|  |  |  |  |  |  | Duration: 4 weeks |  |  |  |  |
| 8 | Correia et al., 2023 | RCT | N=18, 18/0 | ALL: 23.7±2.6years; / | Recreational athletes with resistance training | F: 3 sessions/week; 4 sets/session; 8-10 repetitions/set | Protocol: 16 (fasting) / 8 (eating) | All participants attended 100% of the training sessions and, as in previous studies, exercise sessions were completed within the feeding window period. | Habitual diet | BM →  FM →  FFM →  CSJ Jump height ↓  SJ Jump height →  Ballistic Strength-Bench press throw mean power → |
|  |  |  |  |  |  | I: 85% of the 1-RM, with 90–120 s of rest between sets and exercises | Eating window: 1:00 PM to 9:00 PM |  |  |  |
|  |  |  |  |  |  | T: / |  |  |  |  |
|  |  |  |  |  |  | T: resistance training |  |  |  |  |
|  |  |  |  |  |  | Duration: 4 weeks |  |  |  |  |
| 9 | Tovar et al., 2021 | RCT | N=15, 15/0 | ALL: 28.7±5.2years; / | Highly trained male runners | Duration: 4 weeks | Protocol: 16 (fasting) / 8 (eating) | Nine subjects did not finish the study due to personal commitments unrelated to the study protocol and three subjects were disqualified after consent. Of these, four subjects were randomized to a diet and dropped later, two were randomized to a control diet and two were randomized to the intervention of TRF. | 12-h normal diet(12-h feeding window) | BMs →  LM →  FM ↓  BF % ↓ |
|  |  |  |  |  |  | Other: Subjects self-selected a personalized 4-week training routine ≥32 km·week−1 | Other: not to request similar meal times for all participants |  |  |  |
| 10 | Moro et al., 2021a | RCT | N=20, 20/0 | / | Recreational athletes with resistance training | F: 3 sessions/week | Protocol: 16 (fasting) / 8 (eating) | Six subjects withdrew during the first month. | Normal meal distribution regimen | BM →  FFM →  FM →  Bench press 1RM →  Leg press 1RM → |
|  |  |  |  |  |  | I: between 75% and 90% of 1RM to alternate strength and hypertrophy training cycles | Eating window: ~1 PM, 4 PM, and 8 PM |  |  |  |
|  |  |  |  |  |  | T: between 4:00 and 6:00 PM |  |  |  |  |
|  |  |  |  |  |  | T: resistance training |  |  |  |  |
|  |  |  |  |  |  | Duration: 8 weeks |  |  |  |  |
| 11 | Moro et al., 2021b | RCT | N=20, 20/0 | / | Recreational athletes with resistance training | F: 3 sessions/week | Protocol: 16 (fasting) / 8 (eating) | Six subjects withdrew during the first month. | Normal meal distribution regimen | BM ↓  FFM ↓  FM ↓  Bench press 1RM →  Leg press 1RM → |
|  |  |  |  |  |  | I: between 75% and 90% of 1RM to alternate strength and hypertrophy training cycles | Eating window: ~1 PM, 4 PM, and 8 PM |  |  |  |
|  |  |  |  |  |  | T: between 4:00 and 6:00 PM |  |  |  |  |
|  |  |  |  |  |  | T: resistance training |  |  |  |  |
|  |  |  |  |  |  | Duration: 12 months |  |  |  |  |
| 12 | Brady et al., 2021 | RCT | N=17, 17/0 | EXP: 35.9±8.6years; / CON: 39.9±3.0years; / | middle- and long-distance runners | Duration: 8 weeks | Protocol: 16 (fasting) / 8 (eating) | Four participants failed to complete the minimum amount of training sessions, one participant was injured, and one participant did not respond to researchers at MID for food diary analysis. | Habitual dietary patterns | BM ↓  FM →  FFM →  VO2peak → |
|  |  |  |  |  |  | Other: Participants were instructed to continue with their habitual training routine for the duration of the intervention | Eating window: 12:00 AM to 8:00 PM |  |  |  |
|  |  |  |  |  |  |  | Other: the dietary intake within the daily eating window was considered to be ad libitum and without caloric restriction. |  |  |  |
| 13 | Correia et al., 2021 | RCT | N=12, 12/0 | ALL: 22.4±2.8years; 24.2±2.0kg/m² | Recreational athletes with resistance training | Duration: 4 weeks | Protocol: 16 (fasting) / 8 (eating) | Among the six participants that were excluded due to lack of adherence. | Usual dietary pattern | BM →  FM →  FM% →  FFM → |
|  |  |  |  |  |  | Other: continue their habitual training throughout the study | Eating window: 1:00 PM to 9:00 PM |  |  |  |
| 14 | Stratton et al., 2020 | RCT | N=26, 26/0 | EXP: 22.9±3.6years; / CON: 22.5±2.2years; / | Recreational athletes with resistance training | F: 3 sessions/week | Protocol: 16 (fasting) / 8 (eating) | One individual was excluded for lack of adherence to the fasting protocol (TRF group), and three were excluded (TRF: 1, ND: 2) for lack of dietary adherence. | Normal daily feeding schedule with a prescribed 25% caloric deficit | BM →  FM →  BF% →  VJ HT →  VJ PP →  Leg press 1RM →  leg press repetitions to failure →  bench press 1RM →  bench press repetitions to failure → |
|  |  |  |  |  |  | I: bench and leg press repetitions ranging from 3 to 8. The bench press weights were based on percentages from the pre-intervention e1RM | Other: a prescribed 25% caloric deficit |  |  |  |
|  |  |  |  |  |  | T: between 3:00 and 8:00 PM |  |  |  |  |
|  |  |  |  |  |  | T: resistance training |  |  |  |  |
|  |  |  |  |  |  | Duration: 4 weeks |  |  |  |  |
| 15 | Moro et al., 2020 | RCT | N=16, 16/0 | EXP: 19.38±2.39years; 21.85±1.65kg/m² CON: 19.38±1.60years; 22.47±1.83kg/m² | elite cyclists | F: 6 sessions/week | Protocol: 16 (fasting) / 8 (eating) | Each participant was strictly supervised by the personal team dietitian which ensured the adherence to diet protocol in terms of feeding window and caloric intake. | Traditional pattern | BM ↓  FM% →  FFM→ |
|  |  |  |  |  |  | I: a mild/medium pace at the steady state | Eating window: breakfast (10–11 a.m.), lunch (1–2 p.m.), dinner (6–7 p.m.) |  |  |  |
|  |  |  |  |  |  | T: between 10:00 AM and 6:00 PM |  |  |  |  |
|  |  |  |  |  |  | T: long distance running |  |  |  |  |
|  |  |  |  |  |  | Duration: 4 weeks |  |  |  |  |
|  |  |  |  |  |  | Other: The training schedule included 500 ± 50 km/week |  |  |  |  |
| 16 | Tinsley et al., 2019 | RCT | N=17, 0/17 | EXP: 22.1±8.98years; / CON: 22.0±7.57years; / | Recreational athletes with resistance training | F: 3 sessions/week (i.e., Mondays, Wednesdays, and Fridays) | Protocol: 16 (fasting) / 8 (eating) | Average group compliance with the assigned protocol was ≥89% for the assigned eating schedule. | Requiring breakfast consumption and self-selected intervals | BM →  FM →  FFM →  BF% →  1RMBP →  RTFBP →  1RMLP →  RTFLP →  VJ→ |
|  |  |  |  |  |  | I: train to momentary muscular exhaustion during each set, and the load was adjusted as necessary to ensure compliance with the specified repetition range | Eating window: 12:00 AM to 8:00 PM |  |  |  |
|  |  |  |  |  |  | T: between 12:00 AM and 1:00 PM |  |  |  |  |
|  |  |  |  |  |  | T: supervised resistance training |  |  |  |  |
|  |  |  |  |  |  | Duration: 8 weeks |  |  |  |  |

* ↑ and ↓ indicate statistically significant between-group differences between TRE and non-TRE conditions, whereas → indicates no significant between-group difference.

## 2.2 Effect of TRE on energy intake, body composition and performance

Table S3 Meta-analysis for this study’s findings

| **Outcome** | **Hedge’s** | ***p-value*** | **[95%CI]** | **Heterogeneity** | |
| --- | --- | --- | --- | --- | --- |
|  |  |  |  | ***I²* (%)** | ***p-value*** |
| Energy intake | -0.35 | 0.11 | (-0.77, 0.07) | 0 | 0.58 |
| Protein | -0.82 | 0.13 | (-1.87, 0.23) | 85.92 | 0.00 |
| Carbohydrate | -0.31 | 0.32 | (-0.93, 0.31) | 63.44 | 0.02 |
| Body mass | -0.27 | 0.02 | (-0.50, -0.04) | 0 | 0.91 |
| Fat mass | -0.23 | 0.07 | (-0.48, 0.02) | 0 | 1.00 |
| Body fat% | -0.39 | 0.01 | (-0.68, -0.11) | 0 | 0.92 |
| Lean mass | -0.16 | 0.21 | (-0.42, 0.09) | 0 | 0.99 |
| Aerobic capacity | 0.04 | 0.82 | (-0.29, 0.37) | 0 | 0.78 |
| Muscular endurance | -0.20 | 0.23 | (-0.52, 0.13) | 0 | 0.75 |
| Muscular strength | 0.01 | 0.94 | (-0.25, 0.27) | 0 | 0.98 |
| Power | -0.12 | 0.47 | (-0.44, 0.20) | 0 | 0.87 |

Table S4 Subgroup analysis results

| **Outcome** | **Variables** | **No. of**  **studies** | **Hedge’s** | ***p-value*** | **[95%CI]** | **Heterogeneity** | |
| --- | --- | --- | --- | --- | --- | --- | --- |
|  |  |  |  |  |  | ***I²* (%)** | ***p-value*** |
| Body mass | Elite athletes | 5 | -0.39 | 0.05 | (-0.78, 0.00) | 0 | 0.65 |
|  | Recreational athletes | 9 | -0.21 | 0.15 | (-0.50, 0.08) | 0 | 0.86 |
| Fat mass | Elite athletes | 3 | -0.29 | 0.26 | (-0.78, 0.21) | 0 | 1.00 |
|  | Recreational athletes | 10 | -0.21 | 0.15 | (-0.50, 0.07) | 0 | 1.00 |
| Body fat% | Elite athletes | 3 | -0.67 | 0.01 | (-1.18, -0.16) | 0 | 0.89 |
|  | Recreational athletes | 7 | -0.27 | 0.13 | (-0.61, 0.08) | 0 | 0.92 |
| Lean mass | Elite athletes | 5 | -0.11 | 0.58 | (-0.52, 0.29) | 0 | 0.96 |
|  | Recreational athletes | 8 | -0.19 | 0.24 | (-0.52, 0.13) | 0 | 0.90 |

Table S5 Sensitivity analysis for this study’s findings

| **Outcome** | **Excluding long-term Moro** | | | **Excluding Blake 2025** | | |
| --- | --- | --- | --- | --- | --- | --- |
|  | **Hedge’s** | ***p-value*** | **[95%CI]** | **Hedge’s** | ***p-value*** | **[95%CI]** |
| Energy intake | -0.23 | 0.34 | (-0.70, 0.24) | -0.35 | 0.11 | (-0.77, 0.07) |
| Protein | -0.72 | 0.10 | (-1.57, 0.13) | -0.82 | 0.13 | (-1.87, 0.23) |
| Carbohydrate | -0.19 | 0.54 | (-0.82, 0.43) | -0.31 | 0.32 | (-0.93, 0.31) |
| Body mass | -0.22 | 0.08 | (-0.47, 0.03) | -0.29 | 0.03 | (-0.54, -0.04) |
| Fat mass | -0.21 | 0.11 | (-0.47, 0.05) | -0.23 | 0.08 | (-0.49, 0.02) |
| Body fat% | -0.39 | 0.01 | (-0.68, -0.11) | -0.41 | 0.01 | (-0.71, -0.11) |
| Lean mass | -0.11 | 0.42 | (-0.37, 0.15) | -0.18 | 0.18 | (-0.44, 0.08) |
| Aerobic capacity | 0.04 | 0.82 | (-0.29, 0.37) | 0.04 | 0.82 | (-0.29, 0.37) |
| Muscular endurance | -0.20 | 0.23 | (-0.52, 0.13) | -0.30 | 0.13 | (-0.69, 0.09) |
| Muscular strength | -0.05 | 0.72 | (-0.35, 0.24) | 0.04 | 0.82 | (-0.27, 0.34) |
| Power | -0.12 | 0.47 | (-0.44, 0.20) | -0.12 | 0.47 | (-0.44, 0.20) |

Table S6 Outcome-specific robust variance estimation sensitivity analysis

| **Outcome** | **K effect sizes** | **K cohorts** | **RVE Hedge’s g [95% CI]** | **Adjusted df** | **RVE *p*** |
| --- | --- | --- | --- | --- | --- |
| Energy intake | 5 | 4 | -0.31 (-0.92, 0.30) | 2.977 | 0.21 |
| Protein | 6 | 4 | -0.66 (-2.21, 0.88) | 2.979 | 0.27 |
| Carbohydrate | 6 | 4 | -0.40 (-1.54, 0.75) | 2.996 | 0.35 |
| Body mass | 14 | 13 | -0.25 (-0.43, -0.07) | 11.192 | 0.01 |
| Fat mass | 13 | 12 | -0.22 (-0.32, -0.12) | 10.025 | < 0.001 |
| Body fat% | 10 | 10 | NA | NA | NA |
| Lean mass | 13 | 12 | -0.14 (-0.27, -0.01) | 10.053 | 0.03 |
| Aerobic capacity | 8 | 4 | 0.02 (-0.32, 0.35) | 2.993 | 0.88 |
| Muscular endurance | 7 | 4 | -0.20 (-0.64, 0.24) | 2.880 | 0.23 |
| Muscular strength | 11 | 4 | -0.01 (-0.21, 0.18) | 2.899 | 0.84 |
| Power | 7 | 4 | -0.20 (-0.73, 0.34) | 2.890 | 0.33 |

*NA, RVE not applicable because no within-cohort multiplicity was present. Estimates with adjusted df <4 should be interpreted cautiously

Table S7 GRADE level of evidence for this study’s findings

| **Outcome** | **Risk of bias** | **Inconsistency** | **Indirectness** | **Imprecision** | **Publication bias** | **Certainty** |
| --- | --- | --- | --- | --- | --- | --- |
| Energy intake | Seriousᵃ | Not serious | Not serious | Seriousᶜ | Not suspectedᵉ | ⨁⨁◯◯ Low |
| Protein | Seriousᵃ | Seriousᵇ | Not serious | Seriousᶜ | Not suspectedᵉ | ⨁◯◯◯ Very low |
| Carbohydrate | Seriousᵃ | Seriousᵇ | Not serious | Seriousᶜ | Not suspectedᵉ | ⨁◯◯◯ Very low |
| Body mass | Seriousᵃ | Not serious | Not serious | Seriousᵈ | Not suspectedᵉ | ⨁⨁◯◯ Low |
| Fat mass | Seriousᵃ | Not serious | Not serious | Seriousᶜ | Not suspectedᵉ | ⨁⨁◯◯ Low |
| Body fat% | Seriousᵃ | Not serious | Not serious | Not serious | Not suspectedᵉ | ⨁⨁⨁◯ Moderate |
| Lean mass | Seriousᵃ | Not serious | Not serious | Seriousᶜ | Not suspectedᵉ | ⨁⨁◯◯ Low |
| Aerobic capacity | Seriousᵃ | Not serious | Not serious | Seriousᶜ | Not suspectedᵉ | ⨁⨁◯◯ Low |
| Muscular endurance | Seriousᵃ | Not serious | Not serious | Seriousᶜ | Not suspectedᵉ | ⨁⨁◯◯ Low |
| Muscular strength | Seriousᵃ | Not serious | Not serious | Seriousᶜ | Not suspectedᵉ | ⨁⨁◯◯ Low |
| Power | Seriousᵃ | Not serious | Not serious | Seriousᶜ | Not suspectedᵉ | ⨁⨁◯◯ Low |

ᵃ Risk of bias: Downgraded because several contributing studies had some concerns or high risk of bias.
ᵇ Inconsistency: Downgraded because of substantial heterogeneity.
ᶜ Imprecision: Downgraded because the CI crossed the null and/or few independent cohorts were available.
ᵈ Imprecision: Downgraded because the body-mass effect was sensitive to exclusion of the long-term Moro assessment.
ᵉ Publication bias: No downgrade; formal asymmetry testing was limited by the small number of studies.

Figure S1

|   Body mass |   Fat mass |
| --- | --- |
|   Body fat% |   Lean mass |
|   Muscular strength |  |

Fig S1. The funnel plot of the effect of TRE on body composition and muscular strength

## 3 PRISMA 2020 Checklist

| **Section and Topic** | **Item #** | **Checklist item** | **Location where item is reported** |
| --- | --- | --- | --- |
| **TITLE** | | |  |
| Title | 1 | Identify the report as a systematic review. | Title |
| **ABSTRACT** | | |  |
| Abstract | 2 | See the PRISMA 2020 for Abstracts checklist. | Abstract |
| **INTRODUCTION** | | |  |
| Rationale | 3 | Describe the rationale for the review in the context of existing knowledge. | Introduction |
| Objectives | 4 | Provide an explicit statement of the objective(s) or question(s) the review addresses. | Introduction |
| **METHODS** | | |  |
| Eligibility criteria | 5 | Specify the inclusion and exclusion criteria for the review and how studies were grouped for the syntheses. | Data sources, searches, and study selection |
| Information sources | 6 | Specify all databases, registers, websites, organisations, reference lists and other sources searched or consulted to identify studies. Specify the date when each source was last searched or consulted. | Data sources, searches, and study selection |
| Search strategy | 7 | Present the full search strategies for all databases, registers and websites, including any filters and limits used. | Data sources, searches, and study selection |
| Selection process | 8 | Specify the methods used to decide whether a study met the inclusion criteria of the review, including how many reviewers screened each record and each report retrieved, whether they worked independently, and if applicable, details of automation tools used in the process. | Data sources, searches, and study selection |
| Data collection process | 9 | Specify the methods used to collect data from reports, including how many reviewers collected data from each report, whether they worked independently, any processes for obtaining or confirming data from study investigators, and if applicable, details of automation tools used in the process. | Data extraction, outcomes, and risk of bias assessment |
| Data items | 10a | List and define all outcomes for which data were sought. Specify whether all results that were compatible with each outcome domain in each study were sought (e.g. for all measures, time points, analyses), and if not, the methods used to decide which results to collect. | Data extraction, outcomes, and risk of bias assessment |
|  | 10b | List and define all other variables for which data were sought (e.g. participant and intervention characteristics, funding sources). Describe any assumptions made about any missing or unclear information. | Data extraction, outcomes, and risk of bias assessment |
| Study risk of bias assessment | 11 | Specify the methods used to assess risk of bias in the included studies, including details of the tool(s) used, how many reviewers assessed each study and whether they worked independently, and if applicable, details of automation tools used in the process. | Data extraction, outcomes, and risk of bias assessment |
| Effect measures | 12 | Specify for each outcome the effect measure(s) (e.g. risk ratio, mean difference) used in the synthesis or presentation of results. | Statistical analysis and grading the evidence |
| Synthesis methods | 13a | Describe the processes used to decide which studies were eligible for each synthesis (e.g. tabulating the study intervention characteristics and comparing against the planned groups for each synthesis (item #5)). | Statistical analysis and grading the evidence |
|  | 13b | Describe any methods required to prepare the data for presentation or synthesis, such as handling of missing summary statistics, or data conversions. | Statistical analysis and grading the evidence |
|  | 13c | Describe any methods used to tabulate or visually display results of individual studies and syntheses. | Statistical analysis and grading the evidence |
|  | 13d | Describe any methods used to synthesize results and provide a rationale for the choice(s). If meta-analysis was performed, describe the model(s), method(s) to identify the presence and extent of statistical heterogeneity, and software package(s) used. | Statistical analysis and grading the evidence |
|  | 13e | Describe any methods used to explore possible causes of heterogeneity among study results (e.g. subgroup analysis, meta-regression). | Statistical analysis and grading the evidence |
|  | 13f | Describe any sensitivity analyses conducted to assess robustness of the synthesized results. | Statistical analysis and grading the evidence |
| Reporting bias assessment | 14 | Describe any methods used to assess risk of bias due to missing results in a synthesis (arising from reporting biases). | Statistical analysis and grading the evidence |
| Certainty assessment | 15 | Describe any methods used to assess certainty (or confidence) in the body of evidence for an outcome. | Statistical analysis and grading the evidence |
| **RESULTS** | | |  |
| Study selection | 16a | Describe the results of the search and selection process, from the number of records identified in the search to the number of studies included in the review, ideally using a flow diagram. | Results and Fig. 1 |
|  | 16b | Cite studies that might appear to meet the inclusion criteria, but which were excluded, and explain why they were excluded. | Results and Fig. 1 |
| Study characteristics | 17 | Cite each included study and present its characteristics. | Participant characteristics and Table S2 |
| Risk of bias in studies | 18 | Present assessments of risk of bias for each included study. | Risk of bias and Fig. 2 |
| Results of individual studies | 19 | For all outcomes, present, for each study: (a) summary statistics for each group (where appropriate) and (b) an effect estimate and its precision (e.g. confidence/credible interval), ideally using structured tables or plots. | Meta-analysis |
| Results of syntheses | 20a | For each synthesis, briefly summarise the characteristics and risk of bias among contributing studies. | Meta-analysis |
|  | 20b | Present results of all statistical syntheses conducted. If meta-analysis was done, present for each the summary estimate and its precision (e.g. confidence/credible interval) and measures of statistical heterogeneity. If comparing groups, describe the direction of the effect. | Meta-analysis |
|  | 20c | Present results of all investigations of possible causes of heterogeneity among study results. | Meta-analysis |
|  | 20d | Present results of all sensitivity analyses conducted to assess the robustness of the synthesized results. | Meta-analysis |
| Reporting biases | 21 | Present assessments of risk of bias due to missing results (arising from reporting biases) for each synthesis assessed. | Meta-analysis |
| Certainty of evidence | 22 | Present assessments of certainty (or confidence) in the body of evidence for each outcome assessed. | GRADE assessment and sensitivity analyses and Table S7 |
| **DISCUSSION** | | |  |
| Discussion | 23a | Provide a general interpretation of the results in the context of other evidence. | Discussion |
|  | 23b | Discuss any limitations of the evidence included in the review. | Discussion |
|  | 23c | Discuss any limitations of the review processes used. | Discussion |
|  | 23d | Discuss implications of the results for practice, policy, and future research. | Discussion |
| **OTHER INFORMATION** | | |  |
| Registration and protocol | 24a | Provide registration information for the review, including register name and registration number, or state that the review was not registered. | Methods |
|  | 24b | Indicate where the review protocol can be accessed, or state that a protocol was not prepared. | Methods |
|  | 24c | Describe and explain any amendments to information provided at registration or in the protocol. | Methods |
| Support | 25 | Describe sources of financial or non-financial support for the review, and the role of the funders or sponsors in the review. | Funding information |
| Competing interests | 26 | Declare any competing interests of review authors. | Competing interest |
| Availability of data, code and other materials | 27 | Report which of the following are publicly available and where they can be found: template data collection forms; data extracted from included studies; data used for all analyses; analytic code; any other materials used in the review. | Data availability |

*From:* Page MJ, McKenzie JE, Bossuyt PM, Boutron I, Hoffmann TC, Mulrow CD, et al. The PRISMA 2020 statement: an updated guideline for reporting systematic reviews. BMJ 2021;372:n71. doi: 10.1136/bmj.n71. This work is licensed under CC BY 4.0. To view a copy of this license, visit <https://creativecommons.org/licenses/by/4.0/>
